# Effect of age on chronic inflammation and responsiveness to bacterial and viral challenges

**DOI:** 10.1371/journal.pone.0188881

**Published:** 2017-11-29

**Authors:** Ingrid Elisia, Vivian Lam, Elyse Hofs, Michael Yu Li, Mariah Hay, Brandon Cho, Angela Brooks-Wilson, Miriam Rosin, Luke Bu, William Jia, Gerald Krystal

**Affiliations:** 1 The Terry Fox Laboratory, British Columbia Cancer Agency, Vancouver, British Columbia, Canada; 2 Genome Sciences Centre, British Columbia Cancer Agency, Vancouver, British Columbia, Canada; 3 Department of Biomedical Physiology and Kinesiology, Simon Fraser University, Burnaby, British Columbia, Canada; 4 Cancer Control Research, British Columbia Cancer Agency, Vancouver, British Columbia, Canada; 5 Brain Research Centre, University of British Columbia, Vancouver, British Columbia, Canada; Purdue University, UNITED STATES

## Abstract

To identify reliable biomarkers of age-related changes in chronic inflammation and responsiveness to bacterial and viral challenges, we evaluated endogenous and *ex vivo* stimulated levels of 18 inflammatory markers, using whole blood collected in EDTA and sodium heparin tubes from 41 healthy volunteers, i.e., 11 men + 10 women aged 20–35 and 10 men + 10 women aged 50–77. These studies revealed significant differences in the levels of inflammatory markers when blood was collected in EDTA versus sodium heparin and age related differences in these biomarkers were confirmed with blood collected in EDTA from 120 healthy volunteers in 3 age categories, ie, 20 men + 20 women, aged 20–35, 36–49 and 50–77. Studies with unstimulated blood samples, to measure levels of chronic inflammation, revealed a significant increase with age in IL-12p70, CRP and PGE_2_, consistent with the concept of “inflammaging”, and a decrease in G-CSF in both men and women. Interestingly, in response to *E*. *coli* stimulation, PGE_2_ levels were markedly reduced in the 50–77 year old cohort while they were increased following Herpes Simplex virus-1 (HSV-1) stimulation, along with IL-8. In addition, unlike *E*. *coli*, HSV-1 potently stimulated IFNα production, but levels were dramatically reduced in the older cohort, consistent with a reduced ability to generate an anti-viral response. We also found platelets and CD8^+^ T cells were reduced with age while CD4^+^ T cells were significantly increased, resulting in a substantially higher CD4/CD8 ratio in the older cohort. Surprisingly, however, we found that the older cohort exhibited more T cell proliferation and IFNγ production in response to anti-CD3+anti-CD28 stimulation. Importantly, there was considerable person-to-person variation in these inflammatory markers in all age groups, making possible comparisons between a person’s “inflammage” and chronological age. These assays should help to identify individuals at high risk of autoimmune disorders and cancer.

## Introduction

Human aging is characterized by a gradual increase in sub-clinical chronic inflammation (CI), a phenomenon referred to as “inflammaging” [1–5]. This increase in CI with age has been attributed, in part, to the accumulation of senescent cells that secrete pro-inflammatory cytokines and to debris from damaged cells that triggers the activation of macrophages and other innate immune cells [2,6]. Also contributing to this increase in CI is immunosenescence, gradual aging of the immune system that leads to poorer resolution of immune activation [6,7]. In addition to this increase in CI there is substantial evidence of a reduced ability to mount an efficient innate and adaptive immune response to newly encountered pathogens or vaccine antigens as we age [7]. This combination of an increase in low grade CI and a reduced ability to combat foreign or endogenous danger signals is thought to be responsible for many cancers [8–10] as well as a host of other debilitating diseases including atherosclerosis, Alzheimer’s disease and inflammatory bowel disease (IBD) [11].

Importantly, the literature suggests that there is tremendous age-related person-to-person variation in both basal levels of pro- and anti-inflammatory cytokines/chemokines and the levels of these proteins in response to microbial challenge [12]. It would be of great benefit to identify individuals with high levels of CI, i.e., high basal levels of pro-inflammatory cytokines/chemokines, and a reduced ability to eliminate foreign microbes or aberrant cells. Single nucleotide polymorphisms (SNPs) have been shown to have major effects on regulation of the immune system. For example, while *H*. *Pylori* infection is very common, only about 1% of infected people develop gastric cancer; this is due in large part to SNPs in the interleukin (IL)-1β gene that result in elevated IL-1β expression [13,14]. As well, the T_H_17 pathway, which is regulated by IL-23, has been strongly implicated in IBD; SNPs in the IL-23R gene have been identified as risk factors for this disorder [15]. However, while genotyping specific SNPs may yield some insights into a person’s predisposition for CI, it will not reveal a person’s actual level of CI or anti-microbial capability since many other variables come into play such as injuries, alcohol and tobacco use, exercise [11,16], age [17], diet and stress levels [8,10,18] as well as the composition of gut-associated commensal bacteria (i.e., the microbiome) [19,20].

In contrast to CI, which promotes cancer, acute inflammation appears to lead to tumor regression, especially if it triggers a robust T_H_1 response [21,22]. For example, the injection of interleukin-12 (IL-12), a T_H_1 promoting cytokine directly into tumor sites has been shown to markedly reduce tumor burden in mice by skewing tumor-associated macrophages (TAMs) from an M2-like to an M1-like phenotype [23,24]. There are also many mouse and some recent human studies demonstrating that T_H_1-skewing Toll-like receptor (TLR) agonists (e.g., unmethylated CpG DNA, dsRNA) often reduce tumor burden [25,26]. This suggests that people capable of generating a robust T_H_1 response (e.g., upon viral challenge) might have a reduced risk of cancer by killing tumor cells at very early stages.

Identification of individuals who are at high risk of developing cancer and other CI-induced disorders is of value in efforts to prevent such diseases. We optimized assays that give insight into a person’s immune status and used these assays to investigate the effect of age on both the level of CI and response to *ex vivo* challenge with bacteria and viruses using whole blood from healthy volunteers.

## Materials and methods

### Human subjects and blood collection

In our first set of experiments, 41 healthy volunteers were recruited, i.e., 11 men + 10 women aged 20–35 and 10 men + 10 women aged 50–77. Blood was collected into one 6 mL EDTA Vacutainer tube (cat. no. 367861, BD, Mississauga, ON) and one endotoxin-free [27] 10 ml sodium heparin Vacutainer tube (cat. no. 366480, BD, Mississauga, ON). To confirm our findings from this first study, blood was collected in EDTA from 120 healthy volunteers, i.e., 20 men + 20 women aged 20–34, 35–49 and 50–77. All participants gave informed written consent to participate in these studies, which were reviewed and approved by the joint Clinical Research Ethics Board of the University of British Columbia and the BC Cancer Agency (#H12-00727). All subjects were non-smokers with no history of heart disease, dementia, IBD or cancer and had BMIs ranging from 18 to 35, with the youngest cohort having a mean BMI ± SEM of 22.1 ± 0.6 and the oldest cohort, 25.1 ± 0.7). All volunteers were asked to refrain from consuming non-steroidal anti-inflammatory drugs for 2 days and to fast overnight prior to blood draw. All blood samples were collected by trained phlebotomists at the BC Cancer Agency between 8:30 am and 10:00 am to avoid reported changes in cytokine secretion with diurnal rhythms [28].

### Human blood assay

Human blood samples collected in EDTA or sodium heparin containing glass tubes, were mixed gently, kept at 23°C and aliquoted within 2 hrs of collection into 96-well round bottom tissue culture plates. 50 μL of blood was added to individual wells along with 10 μL of either PBS (Control), *Escherichia coli* (*E*. *coli*, One Shot INV 110, Life Technologies, Burlington, ON) at a final concentration of 2 x 10^4^ cells/mL, or Herpes simplex virus-1 (HSV-1) G207 at a multiplicity of infection (MOI) of 0.06 (relative to total white blood cell numbers). Plates were then incubated for 7 hrs in a 5% oxygen, humidified incubator at 37°C. Following incubation, 100 μL of PBS was added to each well, the cells were then thoroughly resuspended and centrifuged at 424 x *g* at 4°C for 5 min. Supernatants were collected and immediately frozen at -80°C.

### Blood differential counts

Blood differential cell counts were carried out on fresh whole blood collected in EDTA rather than heparin tubes to avoid heparin-induced aggregation of platelets [29], using a Coulter Ac•T diff2^TM^ Hematology Analyzer (Beckman-Coulter Corp., Miami, FL).

### Immunophenotyping

Human peripheral blood mononuclear cells (PBMCs) were isolated from heparinized whole blood by density gradient centrifugation with Lymphoprep (StemCell Technologies, Vancouver, BC). The PBMCs were stained with GhostDye Violet 450 viability dye (Tonbo Biosciences, San Diego, CA) for 30 min at 4°C, washed once with PBS containing 2% FBS and 0.05% sodium azide (PFN), and blocked with anti-human CD32 Clone IV.3 (StemCell Technologies, Vancouver, BC) for 15 min at 23°C. This was followed by staining of cell surface markers for 30 min at 23°C. The cells were then washed twice and resuspended in PFN followed by flow cytometric analysis. To identify regulatory T cells, cells were fixed and permeabilized using the FoxP3 Staining Buffer Set (eBioscience, San Diego, CA). The cells were stained with the FoxP3 antibody overnight at 4°C, washed once with PFN and analyzed by flow cytometry. All analysis was performed using a BD LSR Fortessa flow cytometer (BD Biosciences) and data analysis was performed using FlowJo software V10.2 (FlowJo, Ashland, OR). The antibodies used were: CD14-PE (clone MØp9), CD8-PE (clone SK1) and CD3-FITC (clone SK7) from StemCell Technologies, Vancouver, BC; CD45-FITC (Hle1), CD28-APC (clone CD28.2), CD4-PE-Cy7 (clone SK3), CD25-BB515 (clone 2A3), CD127-AF647 (clone HIL-7R-M21) and FoxP3-PE (clone 236A/E7) from BD Biosciences, Mississauga, ON; CD56-APC (clone CMSSB) from eBioscience, San Diego, CA.

### CD3 and CD28 activation

PBMCs isolated as above were counted using a Vi-Cell XR cell viability analyzer (Beckman Coulter, Brea, CA) and resuspended at 10^6^ cells/mL in RPMI + 10% autologous plasma + 100 U/mL penicillin/streptomycin. The PBMCs were aliquoted (50 μl/well) into 96-well flat-bottom tissue culture plates that were pre-coated overnight with 0.5 μg/mL of anti-human CD3 (clone OKT3, eBioscience, San Diego, CA). Anti-human CD28 (clone CD28.2, eBioscience, San Diego, CA) at a final concentration of 2 μg/mL was then added to each well, and the plates incubated in a humidified incubator at 5% CO_2_, 37°C for 4 days. The plates were then centrifuged at 300 x g in a Beckman TJ-6 centrifuge for 5 min and the supernatants collected for IFNγ analysis. Identical plates were pulse-labeled with [methyl-^3^H]-thymidine (1 μCi/well, 2 Ci/mmol, PerkinElmer, Woodbridge, ON) for 24 hrs. Cells were harvested and the incorporated ^3^H-thymidine quantified using a LKB Betaplate Harvester and Liquid Scintillation Counter (LKB Wallac).

### Luminex analysis

A custom magnetic Luminex assay panel from Life Technologies was used to assess the levels of the following 15 cytokines and chemokines in human plasma: IL-1β, G-CSF, IL-10, IL-13, IL-6, IL-17, MIP1α, VEGF, IFNγ, IL-12p70, IFNα, IL-1RA, TNFα, IL-4 and IL-8. Frozen samples were thawed and spun before testing (1000 x g at 4°C for 10 min). Plasma samples were incubated with antibody beads overnight at 4°C. Antibody beads, biotinylated antibodies and streptavidin-RPE were used at ½ of the manufacturer’s recommended amounts. Assay plates were read using a BioPlex 100 instrument utilizing Bio-Plex Manager 6.0 software (Bio-Rad Laboratories, Mississauga, ON).

### PGE_2_, CRP and TGF-β1 measurement

ELISAs for PGE_2_ (Cat #514010, Cayman Chemical Company, Ann Arbor, MI), C-reactive protein (CRP) (Cat # DCRP00, R&D Systems, Minneapolis, MN) and TGF-β1 (Cat # 88–8350, eBioscience, San Diego, CA) were performed according to manufacturer’s instructions. Total TGF-β1 was measured following acid activation using 1N HCl and neutralization of plasma samples.

### Statistical analysis

Significant differences between the means of the cytokine/chemokine levels in the 2 different age groups and between men and women at time zero (i.e., to measure levels of CI), and in *E*. *coli* or HSV-stimulated blood samples were evaluated using a two way ANOVA. When a significant difference in one of the main factors (age or sex) was identified, a Kruskall Wallis test followed by Dunn`s multiple comparison test or Mann-Whitney test was performed in Graphpad Prism 7 to identify significant differences in the expression of cytokine/chemokine levels between the levels of the main factor. In addition, a correlation analysis between cytokines/chemokines and age was performed using Graphpad Prism 7.

## Results

### Optimization of inflammaging assays

Before embarking on studies to compare the levels of CI (at zero time values) and immune responsiveness to bacterial, viral and T cell challenges in young and old healthy volunteers, we set out to optimize our assays. Although both PBMCs and whole blood samples have been used in the literature [30–35], we chose to use whole blood samples since they have been shown to more closely mimic *in vivo* conditions [34], and had the advantages of fewer manipulations and presence of autologous plasma (which likely affects responses to *ex vivo* challenges), granulocytes and red blood cells [36]. To avoid the effects of diurnal rhythms [28] and diet [37] on cytokine secretion, all blood samples were taken between 8:30 am–10:00 am from overnight-fasted volunteers. All volunteers were asked to not take anti-inflammatories and avoid vigorous exercise and/or weight training for 2 days prior to giving blood since the latter has been shown to elevate plasma TNFα [16] and IL-6 [38] levels. We also incubated our blood samples *in vitro* for only 7 hrs since we found it was the shortest time required to detect both “early” and “late” secreted cytokines like IL-10 (data not shown), reduced the possibility of “drift” from their *in vivo* phenotype and avoided the death of neutrophils and red blood cells that were observed with longer incubations.

To challenge blood samples with agents that most closely mimic *in vivo* infections, intact *E*. *coli* and intact HSV-1 were used. We chose *E*. *coli* and HSV-1 concentrations that, in preliminary experiments, gave IL-6 levels that were approximately half way up the two dose response curves for maximal sensitivity. This is in keeping with Bernstein & Murasko’s finding that supraoptimal concentrations of stimuli tended to mask age-related differences in cytokine levels [30]. To monitor inter-assay variation we also included within every assay PBMCs that were frozen from healthy volunteers and tested with and without *E*. *coli* stimulation. Whole blood samples were incubated in 5% O_2_ rather than standard 20% O_2_ incubators to more closely mimic *in vivo* oxygen and subsequent reactive oxygen species (ROS) levels [39].

After initial testing using the Cytokine Human Magnetic 30-Plex Panel for Luminex from Life Technologies, we selected 15 cytokines/chemokines for analysis based on their previously published roles in CI, T_H_ subtype skewing and detectability following stimulation. In addition, to obtain a more complete picture of inflammatory status we also measured the levels of CRP, total TGF-β1 and PGE_2_ using additional ELISAs since these analytes could not be multiplexed with the custom Luminex panel.

### Differences in chronic inflammation with age

**Fig 1:**
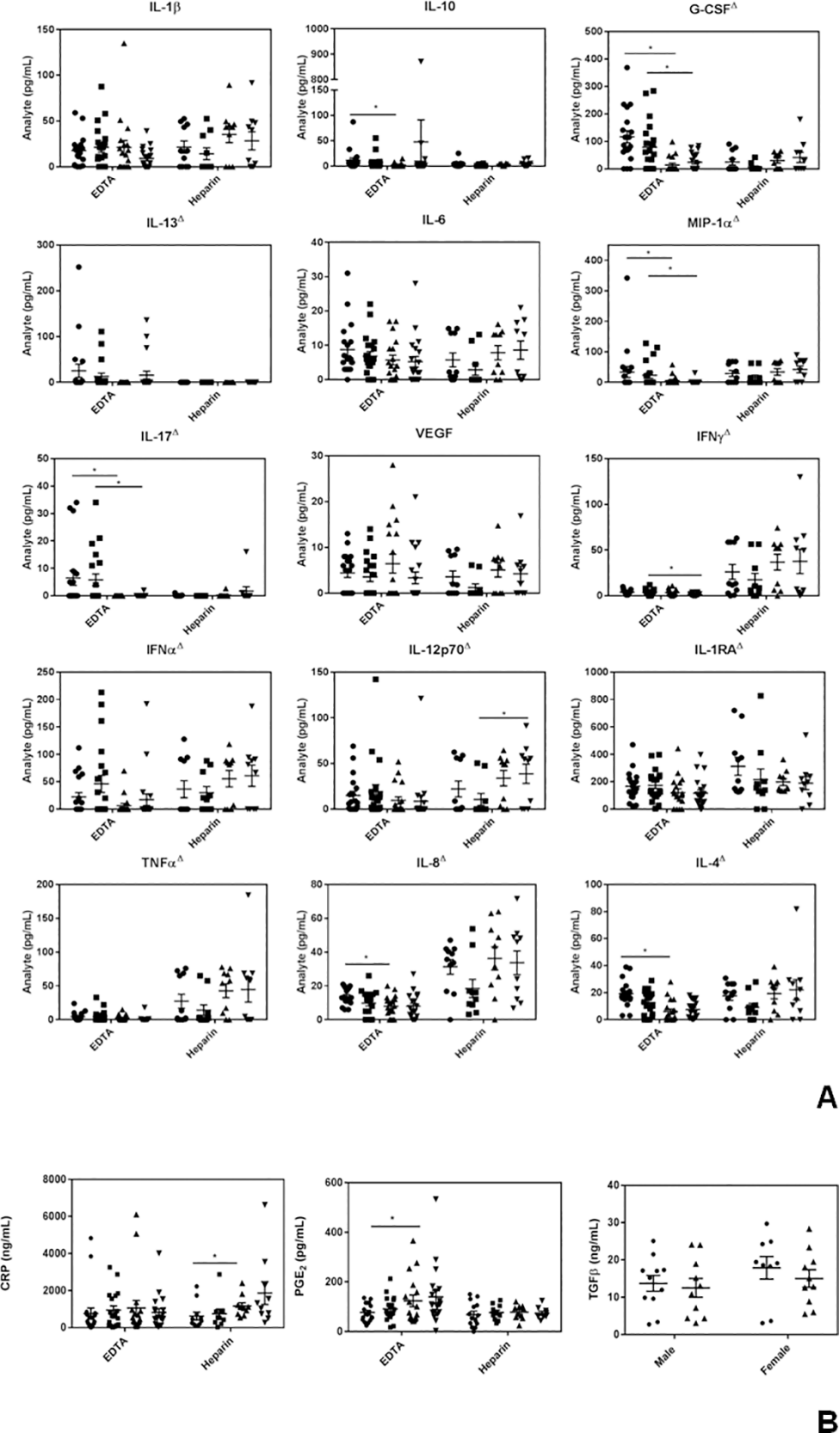
A comparison of endogenous levels of inflammatory markers in fresh blood samples from young and old volunteers collected with EDTA versus heparin. **(A)** Luminex results. ● = males 20–35 years old; ■ = females 20–35 years old; ▲ = males >50 years old and ▼ = females >50 years old. **(B)** CRP, PGE_2_, and total TGFβ levels in zero time (endogenous) samples. For the top 2 panels, ● = males 20–35 years old; ■ = females 20–35 years old; ▲ = males >50 years old and ▼ = females >50 years old. ^Δ^ denotes a significant (P<0.05) difference in cytokine/chemokine levels between EDTA and heparin samples. Results are expressed as the mean ± SEM. * indicates a statistically significant difference (P <0.05) between the young and old cohorts.

To determine the best anti-coagulant to use with our zero time samples (used to assess CI) we compared EDTA and sodium heparin tubes (the latter being pre-screened for endotoxin levels, since some lots of plastic heparin tubes were found to be significantly contaminated, using the Pierce LAL Chromogenic Endotoxin Quantitation kit (cat. no. 88282, Thermo Fisher Scientific). As shown in Fig 1A, of the 15 cytokines/chemokines tested using the Luminex assay on plasma from zero time samples, some analytes showed higher levels with EDTA (G-CSF, IL-13, MIP1α and IL-17) while others showed higher levels with heparin (IFNγ, IFNα, IL-12p70, IL-1RA, TNFα, IL-8 and IL-4). Zero time blood collected in EDTA showed statistically significant, lower levels of G-CSF, MIP1α and IL-17 levels in older individuals, in both men and women. As well, there was a significantly higher level of IL-12p70 in older versus younger women, and a trend, albeit not significant, towards higher levels of IL-1β, IFNγ, IFNα and TNFα in heparin-containing blood from both older men and women, consistent with inflammaging. As far as sex differences are concerned we found lower levels of IFNγ in older females than older males and lower IL-10 and IL-4 in older versus younger males, when blood was collected in EDTA tubes (Fig 1).

Data from the ELISA assays (Fig 1B) revealed that CRP levels were higher in the older cohort, particularly in men, in blood collected in heparin. PGE_2_ gave higher levels with EDTA and was also higher in the older cohort. Total (acid treated) TGF-β1 levels, which in preliminary tests were far more robust with EDTA than heparin (data not shown), showed no significant difference with age or gender (Fig 1B).

**Fig 2:**
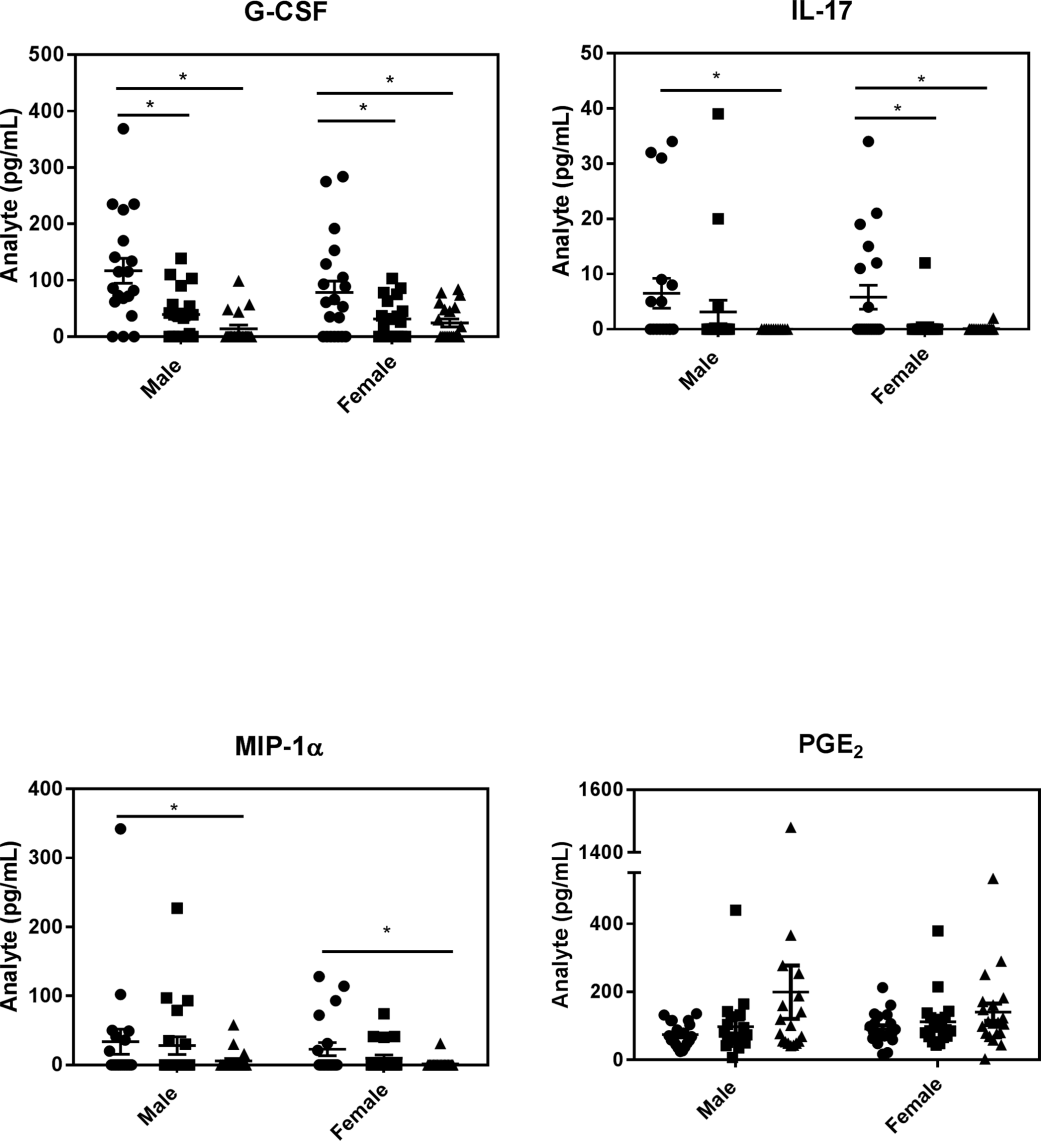
Differences in endogenous levels of IL-17, G-CSF, IL-17, MIP1α and PGE_2_ levels at different ages. Blood collected in EDTA tubes from 20 healthy men + 20 healthy women, aged 20–34 (●), 35–49 (■) and 50–77 (▲) years old and assayed for G-CSF, IL-17, MIP1α and PGE_2_, using Luminex beads and, for PGE_2_ levels, an EIA. Results are expressed as the mean ± SEM. * indicates statistically significant differences (P <0.05) between the different cohorts.

Since zero-time blood collected in EDTA was lower in G-CSF, MIP1α and IL-17 and higher in PGE_2_ in older people, we wanted to confirm and explore these findings in more detail. We thus compared zero time values of these inflammatory markers in blood collected in EDTA from 120 healthy volunteers in 3 different age groups (20 men + 20 women aged 20–34, 35–49 and 50–77). As can be seen in Fig 2, G-CSF, IL-17 and MIP1α were significantly lower in both men and women and PGE_2_ was higher with age, reaching statistical significance only in older men, corroborating our initial results.

### Response to microbial challenge with age

**Fig 3:**
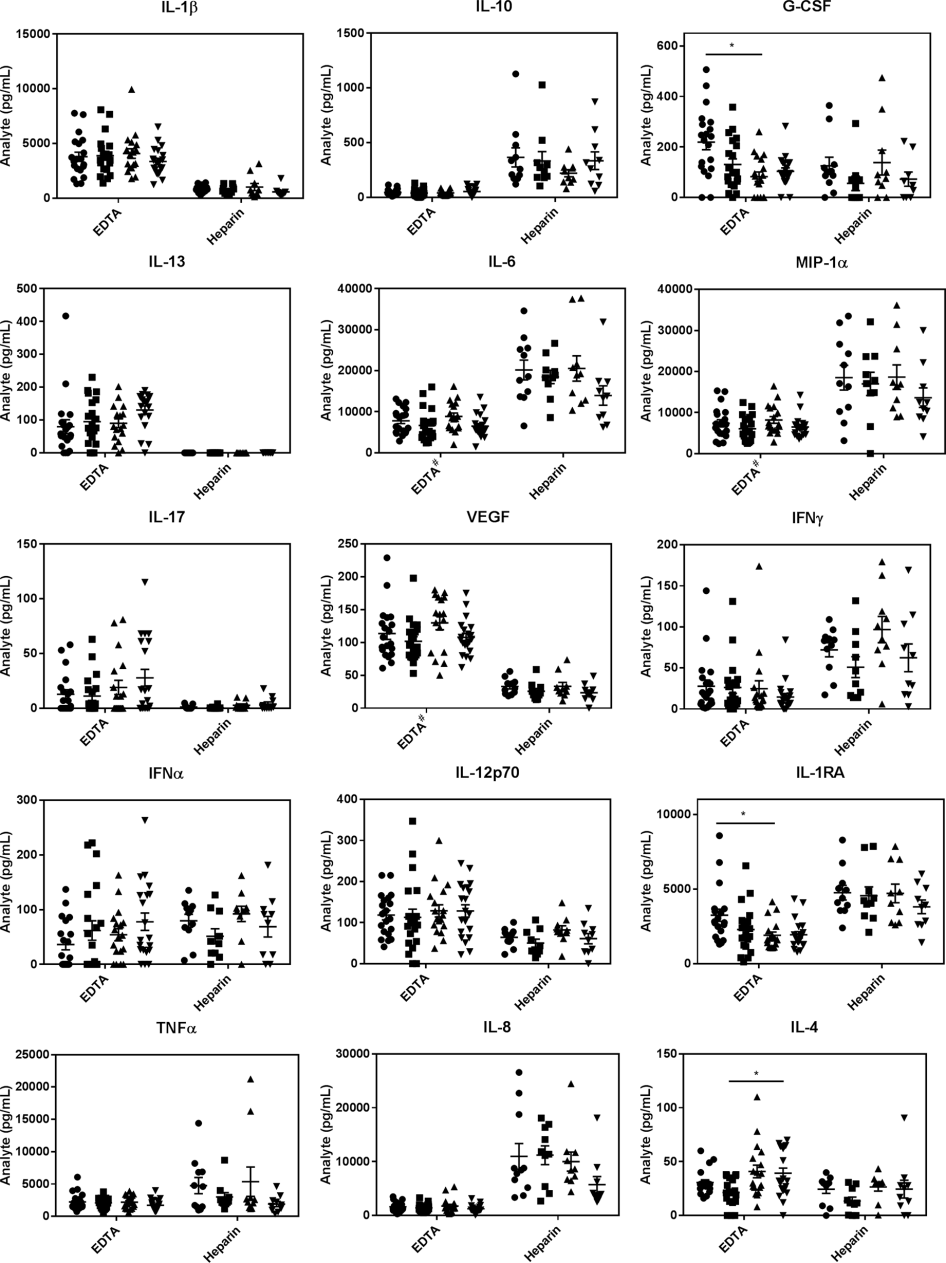
A comparison of *E*. *coli* stimulated levels of inflammatory markers from young and old volunteers collected with EDTA versus heparin. ● = males 20–35 years old; ■ = females 20–35 years old; ▲ = males >50 years old and ▼ = females >50 years old. Results are expressed as the mean ± SEM. * indicates a statistically significant difference (P <0.05) between the young and old cohorts. ^#^ indicates a statistically significant difference (P<0.05) between male and female cohorts.

**Fig 4:**
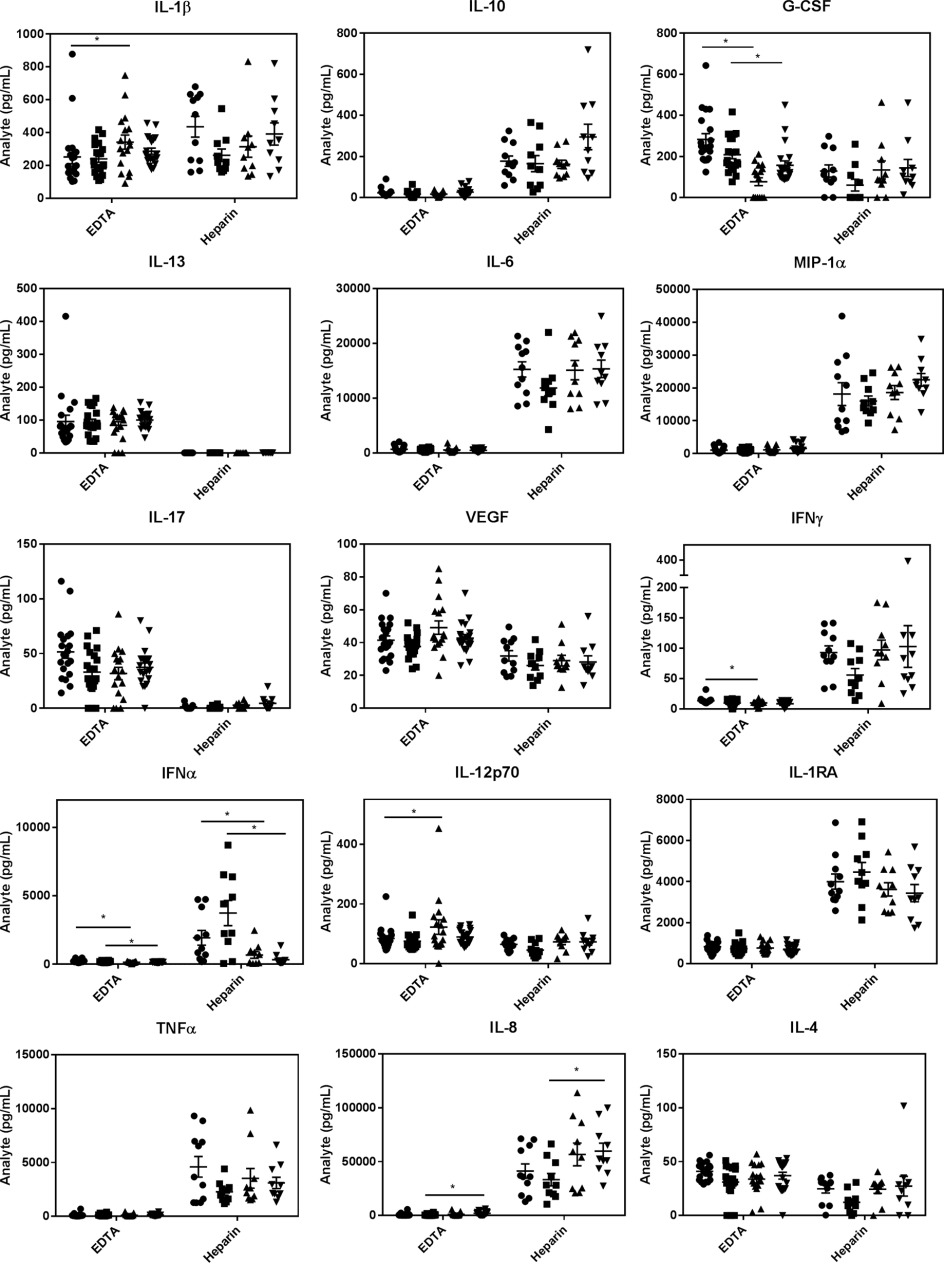
A comparison of HSV-1 stimulated levels of inflammatory markers from young and old volunteers collected with EDTA versus heparin. ● = males 20–35 years old; ■ = females 20–35 years old; ▲ = males >50 years old and ▼ = females >50 years old. Results are expressed as the mean ± SEM. * indicates a statistically significant difference (P <0.05) between the young and old cohorts.

In terms of the best anti-coagulant for *E*. *coli*- or HSV-1-stimulated whole blood samples, we found that heparin tubes gave far more robust cytokine responses for IL-10, IL-6, MIP1α, IFNγ, IL-1RA, TNFα and IL-8, while EDTA gave substantially higher values for IL-1β (*E*. *coli*-induced only), G-CSF, IL-13, IL-17, VEGF, IL-12p70 and IL-4 (Figs 3 and 4). Given the similarity in IL-6 and MIP1α levels triggered by *E*. *coli* and HSV-1, it is interesting that *E*. *coli* stimulated far higher IL-1β (EDTA) than HSV-1 and far lower IL-8 levels (heparin) (compare Figs 3 and 4). Importantly, IFNα was only elevated in response to HSV-1 (not to *E*. *coli*) (compare Figs 3 and 4), consistent with IFNα being a specific anti-viral cytokine [40]. This suggested our blood assays were performing as anticipated and this was substantially more robust when the blood was collected in heparin. Importantly, this cytokine showed a marked drop with age and this statistically significant reduction with age was seen with both men and women and regardless of whether whole blood was collected in heparin or EDTA tubes (Fig 4). As well, similar to zero time values with EDTA, both *E*. *coli*- and HSV-1-stimulated whole blood samples collected in EDTA showed lower G-CSF levels in the older cohort (Figs 3 and 4). However, with *E*. *coli*-challenged samples this difference was only significant in men (Fig 3). These reductions in microbially-stimulated IFNα and G-CSF levels with age were corroborated with EDTA-collected blood samples from our 120 volunteers and showed, once again, that the lower G-CSF in older individuals only reached significance in men (S1 Fig**).** Also worthy of note is that women produced lower levels of IL-6, MIP1α and VEGF than men in response to *E*. *coli* stimulation when blood was collected in EDTA, (Fig 3).

**Fig 5:**
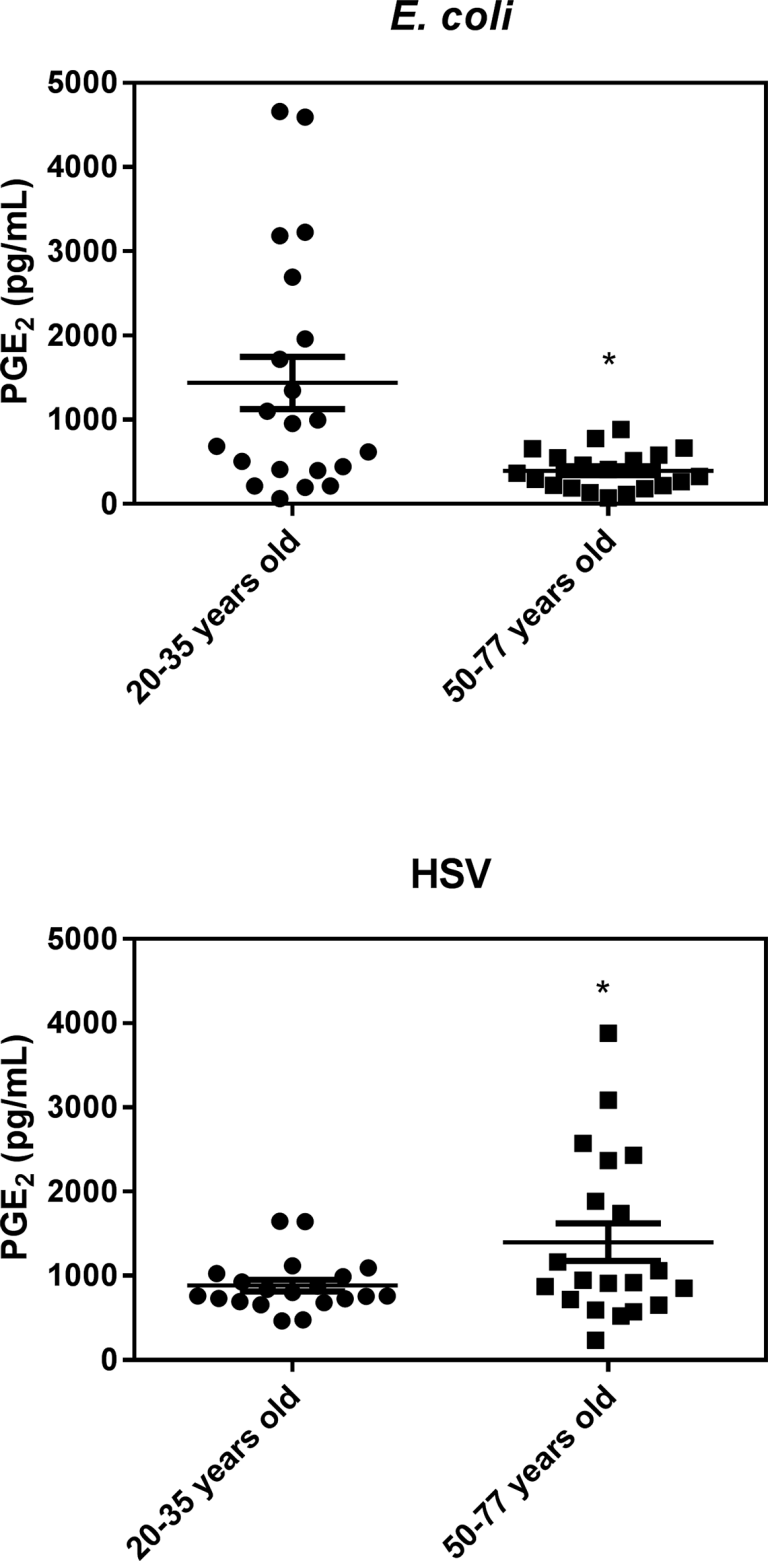
*E coli* and HSV-1 stimulated levels of PGE_2_ in whole blood. Results are expressed as the mean ± SEM.* indicates a statistically significant difference (P <0.05) between the young and old cohort.

Intriguingly, in response to *E*. *coli*-stimulation, we found that PGE_2_ levels were markedly lower in the older cohort while, following HSV-1 stimulation, they were significantly higher (Fig 5). This surprising finding has not been reported previously, to our knowledge, and is of interest because there is a general consensus that PGE_2_ levels increase with age as a result of increased cyclooxygenase in monocytes/macrophages [41,42] and that this inhibits T cell responses [43].

### Effect of age on the adaptive immune system

**Fig 6:**
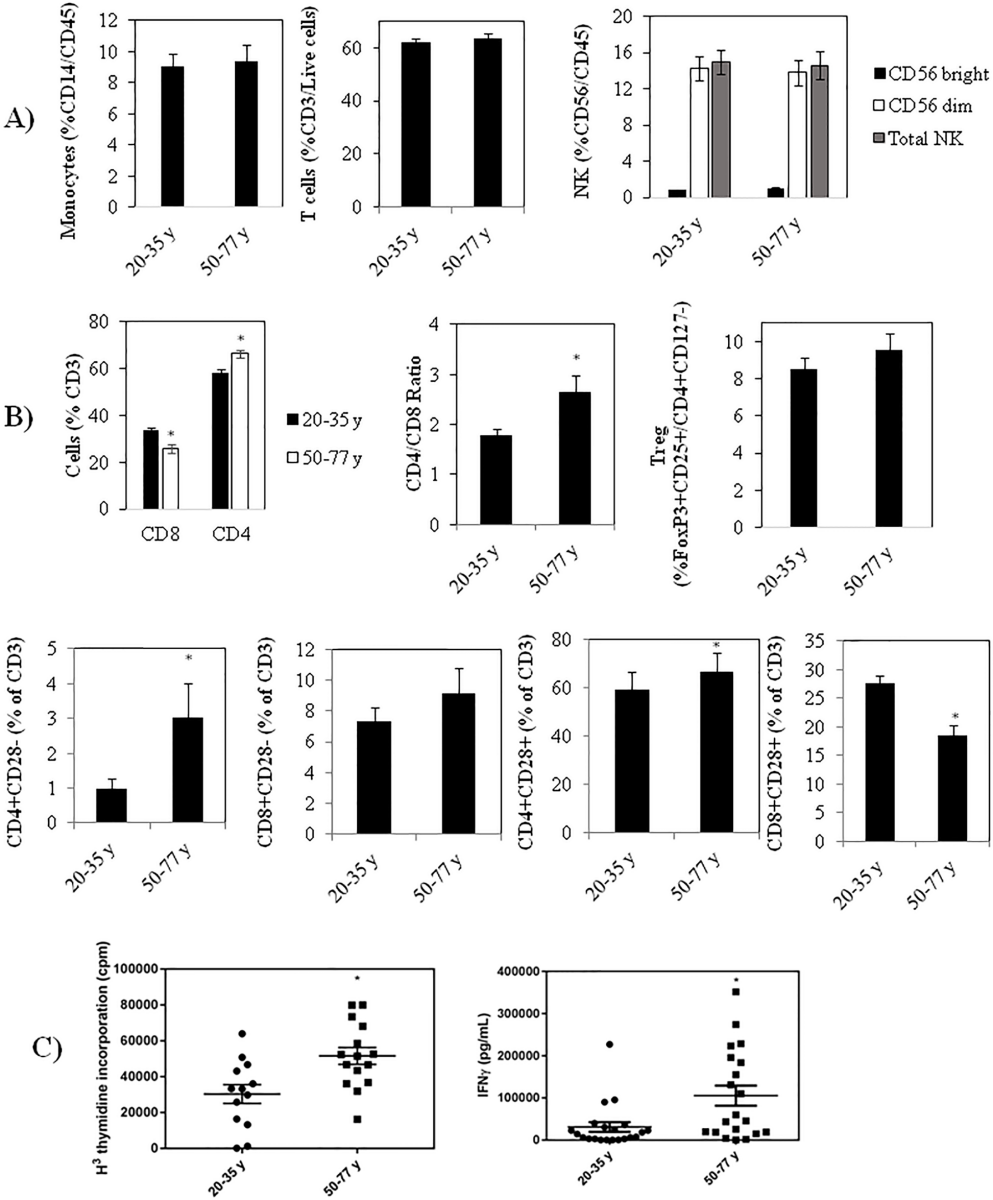
**(A)** Differences in the proportions of monocytes, T cells and NK cells with age. **(B)** Differences with age in the % of T cells that are CD4+ and CD8+, in the CD4/CD8 ratio with age, in the proportion of T cells that are Tregs, and in the proportion of CD4 and CD8 cells that are CD28+ and -. **(C)** Anti-CD3 + anti-CD28 stimulated proliferation (left panel) and IFNγ production (right panel). Results are expressed as the mean ± SEM. * indicates a statistically significant difference (P <0.05) between the young and old cohorts.

To assess the effect of age on the acquired immune system we first carried out blood cell differentials and flow cytometry to look for differences in the proportions of various immune cells in younger and older individuals. Differential analyses on the 41 healthy volunteers revealed lower platelets in the older cohort from an average ± SEM of 208 ± 10 to 175 ± 12 10^9^/L, as has been reported previously [44] but no significant differences in the proportion of monocytes, total natural killer (NK) cells (or NK subsets) or total lymphocytes between the 20–35 and 50–77 year old subjects (Fig 6A and 6B), in contrast to some earlier studies [17]. As well, when we measured specific T cell subsets, we did not find a significant difference in Tregs in the older vs. younger volunteers (Fig 6B), in contrast to Gregg et al [45]. However, we did find a significantly higher proportion of CD4 and lower CD8 cells with age, resulting in a higher CD4/CD8 ratio in the 50–77 year old subjects (Fig 6B), in keeping with previous studies [46]. In addition, while there was a small, but statistically significant increase in the proportion of CD4+CD28- T cells in the older volunteers, no difference in CD8+CD28- T cells was observed between the two age groups.

To evaluate age-related differences in T cell activation, we stimulated PBMCs in 10% autologous plasma with anti-CD3 + anti-CD28 for 4 days and found, surprisingly, that age was associated with a greater cell proliferation and production of IFNγ (Fig 6C). This is in contrast to a number of reports suggesting a lower proliferative response and/or lower IFNγ production in older subjects [47]. It should, however, be noted that many of these earlier studies used less physiologically relevant mitogens (e.g. PHA and concanavalin A) and, when using well-defined stimuli that engage both the T cell receptor and co-stimulatory signals, Sansoni et al found no significant difference in the capability of T cells from centenarians to proliferate compared to young subjects [48]. Our observations are also in agreement with Yen et al (2000) who reported increased IFNγ expression with anti-CD3 + anti-CD28 activated CD4 and CD8 cells from older subjects [47,49]. Our findings are also in keeping with a previous study showing that T cells from older subjects are in a state of increased excitability [50]. It is interesting to note that although we saw a higher number of senescent CD4+CD28- cells, there was still more T cell proliferation upon anti-CD3 + anti-CD28 activation, suggesting that the higher number of senescent cells in the older cohort is not sufficiently large to impact T cell responses.

### Individual variation in inflammaging

To look at person-to-person variation in inflammaging we gave each of the 41 healthy volunteers a unique colored symbol to make possible visualization of all the tested inflammatory markers for each individual (see S2 and S3 Figs). This has allowed us to follow the level of CI of each individual and their response to microbial challenge. For example, a close look at the person with the highest endogenous level of CRP (see last panel, S2 Fig), suggesting a high level of CI, revealed that this woman in the 50–77 age cohort (designated with pink star symbol) had the highest *E*. *coli*-induced IL-RA and highest HSV-1-induced IL-8 levels but nothing else of note. As another example, the two men labeled with blue triangle and pink diamond symbols in the 50–77 year old cohort triggered the highest IL-6, TNFα, IFNγ, G-CSF, VEGF, IL-1β, MIP1α and IL-12p70 levels in response to *E*. *coli* and relatively high levels of these same cytokines, as well as IL-8 and PGE_2_, in response to HSV-1. Of interest, they also triggered very high IL-1RA levels in response to *E*. *coli*. On the other hand, they did not respond at all in terms of HSV-1-induced IFNα production. Interestingly, although they both had very low responses to anti-CD3+ anti-CD28 induced T cell proliferation, pink diamond-labeled volunteer gave the highest and blue triangle-labeled subject the lowest anti-CD3+ anti-CD28-induced production of IFNγ. Also worthy of mention, while the average level of IFNα production in response to HSV-1 was much higher in the younger cohort, there was substantial individual variation in this group and two of the younger women (red diamond and green triangle symbols,) displayed no response at all, suggesting, perhaps, that their inflammage was substantially higher than their chronological age.

## Discussion

There are many contradictory reports in the literature on the effects of age on both levels of CI and levels generated in response to *ex vivo* challenge (e.g., LPS) [17,30,51]. Given that these discrepancies might be due, in part, to differences in the methods used, we evaluated different procedures to optimize both endogenous and microbially-induced pro- and anti-inflammatory cytokine/chemokine levels. Interestingly, we obtained very different results when blood was collected in EDTA versus heparin, in keeping with previous reports showing that, for unchallenged samples, significant differences are observed with different anticoagulants [52,53]. These differences are likely attributable to the fact that (a) EDTA chelates calcium and thus impairs the ability of cells to produce cytokines/chemokines that require Ca^++^-dependent pathways [54] and (b) heparin binds to LPS-binding protein [55] and some cytokines [56,57] and thus has unpredictable effects on cytokine levels [58]. Taken together with our results, this stresses the importance of comparing different anti-coagulants before finalizing procedures for testing specific cytokine/chemokine panels. Another reason for discrepancies in the literature is likely the ages chosen to represent young and old volunteers. Although many aging studies employ an older cohort than the 50–77 year olds we chose, to avoid possible hormonal effects during menopause [30], we specifically wanted to avoid people who might have cytokine differences secondary to frailty [54,59]. In terms of the panel of immune markers selected for this study, we initially chose cytokines/chemokines with well-established roles in microbial infections, T_H_ skewing and/or cancer cell eradication, and then selected a subset that gave detectable levels following microbial stimulation in the majority of samples.

In terms of endogenous measurements, we found no significant differences between levels in non-incubated (0 time) versus 7 hr incubated, unstimulated (control) samples (data not shown), consistent with earlier findings showing no significant cytokine secretion *in vitro* with unstimulated whole blood [12]. As well, for most cytokines/chemokines, we did not detect any significant differences between men and women in either age group, in contrast to earlier studies reporting such differences [60,61]. However, IL-12p70 was found to be significantly higher in older versus younger women while no difference was found in young versus older men. While a number of reports suggest that several pro-inflammatory cytokines are significantly elevated under unstimulated (and LPS stimulated) conditions in older subjects, such as IL-6, TNFα, IL-1β and IL-8 [62–66], other reports suggest no such differences [67]. Our results suggest a significant difference, under unstimulated conditions, in IL-12p70 (females only) and CRP (particularly in men) and only a non-significant trend towards higher levels of IL1β, IFNγ, IFNα and TNFα with age, consistent with the results of Beharka et al who found that when the health status of older subjects was strictly controlled for, using the SENIEUR protocol, no difference in IL-6 was observed in healthy older subjects [68]. It is thus possible that reported increases in these pro-inflammatory cytokines are actually associated with chronic ailments and frailty rather than age itself.

Consistent with one earlier study [67], we observed significantly lower G-CSF levels with age. This cytokine has been shown to stimulate hematopoietic stem cell (HSC) proliferation and subsequent exhaustion [69,70], HSC mobilization into the blood, neutrophil expansion in response to infections and stress granulopoiesis in response to elevated pro-inflammatory cytokines [69]. Its reduction with age may therefore reduce neutrophil expansion and thus resistance to infections but, on the other hand, might also prevent HSC exhaustion. Also of note, G-CSF is produced in response to IL-1β and TNFα and increases pain during arthritic attacks [71]. The higher endogenous CRP (with heparin) that we see with age is controversial, with some reporting increases [72] and others not [73]. Importantly, while we only saw a non-significant correlation between CRP and age (S1 Table) we observed a significant correlation between endogenous CRP levels and BMI. This suggests that our observed increase in CRP with age may be attributable to higher BMIs rather than age *per se*. Of note, CRP has been shown to closely reflect tissue inflammation and may be involved in binding phosphocholine on the surface of pathogens and damaged host cells, activating the classical complement pathway to promote their phagocytosis [74].

The higher endogenous levels of PGE_2_ (with EDTA) we observe with age is also of interest. This lipid signaling molecule has both pro- [75] and anti-inflammatory [76] properties, complicating interpretation of its increase, but it is generally thought of as an inhibitor of both innate and adaptive immunity, in part by increasing MDSCs [76] and Tregs [77]. Compounding its inhibitory effects, it has been reported that the same concentration of PGE_2_ is more effective at inhibiting phytohemagglutinin-stimulated T cell activation in people over 70 years old [43]. An increase with age might be detrimental in terms of combating microbial infections or cancer, or it might be helpful in preventing autoimmune disorders.

In terms of responses to microbial challenges we found that *E*. *coli* stimulated similar levels of all the cytokines/chemokines tested in the young and old cohorts, suggesting that the innate immune response against bacterial infection is mostly preserved with age. As well, we found a significant reduction in PGE_2_ expression from *E*. *coli*-stimulated whole blood from the older cohort. Since this lipid mediator has been shown to increase MDSCs, reduce NK cell activity [76] and inhibit T cell proliferation [78], this may add to the ability of older individuals to retain a strong anti-bacterial response.

With HSV-1 stimulation, on the other hand, PGE_2_ increased with age (Fig 5), along with IL-8. IFNα levels, on the other hand, were dramatically lower in the older cohort following HSV-1 stimulation. Since IL-8 and PGE_2_ are largely expressed by activated monocytes/macrophages, while IFNα is produced primarily by plasmacytoid dendritic cells [79,80], we hypothesize that age may be associated with an enhanced macrophage response, but a dampened NK or dendritic cell response to viral infections. This reduction in dendritic cell response could simply be due to the reduction in plasmacytoid dendritic cells that has been reported to occur with age [79].The increased secretion of IL-8 with age in response to HSV-1 points to an over-reactive innate immune response that may contribute to CI if resolution is not achieved [81]. As well, the age-associated increase in HSV-1-induced PGE_2_, especially when coupled with the dramatic reduction in HSV-1-induced IFNα that we observe with age (which is in agreement with a report by Abb et al [82]), may be responsible for the well documented reduction in the adaptive immune response against viral infections as we age [46,83,84]. Relevant to this and complicating the picture somewhat, while acute type 1 IFN production has been shown to promote viral clearance, in part by enhancing B and T cell responses [85], and prevent tumor formation by increasing p53 [86], chronic type 1 IFN production may actually promote immunosuppression by increasing MDSCs [87].

Pearson correlation analysis of our data (S1–S3 Tables) revealed that many of the cytokines/chemokines that are expressed endogenously or in response to *E*. *coli* or HSV-1 stimulation are correlated. This likely reflects the fact that many cytokines/chemokines are regulated by shared signaling pathways. For example, the *E*. *coli*-induced expression of IL-6 is strongly correlated with that of IL-1β, MIP1α, IL-8 and TNFα, which collectively points to the activation of the NFkB pathway in regulating the transcription of these pro-inflammatory cytokines [88]. On the other hand, the robust expression of IFNα in response to HSV-1 does not correlate with many of the pro-inflammatory cytokines that are upregulated by *E*. *coli* stimulation, confirming the upregulation of virus-specific signaling pathways. Also of interest is that the endogenous expression of cytokines that are typically stimulated in response to *E*. *coli* or HSV-1 (e.g. IL-6, IL-1β, IL-8, TNFα and MIP1α) do not correlate significantly with the levels found in stimulated whole blood samples (data not shown). This suggests that the circulating levels of pro-inflammatory cytokines do not predict the magnitude of a person’s response to a bacterial or vial challenge infection.

In terms of changes in immune cell populations with age, we did not find significant differences in the proportion of monocytes, NK cells or lymphocytes between the 20–35 and 50–77 years old subjects. This is in contrast to earlier studies showing that the myeloid/lymphoid ratio increases with age [17,89]. Consistent with earlier findings [44], however, we did see a decrease in platelets with age. When we investigated specific T cell subsets, we did not find a significantly higher Treg population with age but did find a higher CD4 and a lower CD8 proportion with age, resulting in a higher CD4/CD8 ratio in the 50–77 year old cohort, in keeping with a previous report [46]. In addition, there was a slight but significantly higher proportion of CD4+CD28- T cells in the older volunteers, while no difference in CD8+CD28- T cells was observed between the two age groups. The latter finding contrasts with earlier studies showing an expansion of CD8+CD28- T cells with age [17,90]. However, the difference we observe in CD4+CD28- T cells is consistent with a number of reports suggesting an increase in ‘senescent’ or a highly differentiated CD28- T cells in older subjects [17,84]. Surprisingly, although we observed a higher number of senescent CD4+CD28- cells with age, the older cohort stimulated more T cell proliferation and IFNγ production in response to anti-CD3 + anti-CD28 stimulation, suggesting that the difference in senescent cells in this age group is not sufficiently large to impact intrinsic T cell responses. We thus conclude that T cells in older subjects are capable of robust activation, and that increased susceptibility to viral infections with age [46,83,84] may be due to other aspects of T cell activation. For example, a poor engagement between T cells and antigen presenting cells (APCs), lower levels of T cell stimulators secreted by the innate immune system (eg, IFNα) and increased levels of negative regulators like PGE_2_ [76,77] may reduce T cell activation. Alternatively, as mentioned earlier, it is also possible that the discrepancy in the literature regarding changes associated with aging may be attributable to employing different ages to define ‘old’.

Based on our results, we recommend the minimal panel of inflammatory markers shown in Table 1 to assess inflammaging. In addition, we recommend examining the CD4/CD8 ratio, CD4+CD28- T cell levels and anti-CD3 + anti-CD28 stimulated IFNγ levels (since this is easier to measure than proliferation in many labs and we found that proliferation, in general, correlated with IFNγ production). We also recommend examining the levels of naïve (CD45RA+) and memory (CD45RO+) CD4 and CD8 T cells since both Koch et al [91] and Jackola et al [92] have shown that naïve T cells decrease and memory T cells increase with age, and this likely has a significant impact on T cell responses. Importantly, there was considerable individual variation in these inflammatory markers in both age groups, making possible comparisons between a person’s “inflammage” and their chronological age. Taken together, our results suggest that using robust, reliable assays to measure a person’s inflammage could identify individuals with sub-clinical levels of CI and poor or over-exuberant anti-microbial activity. This information, in turn, may help to identify those at high risk of autoimmune disorders and cancer.

**Table 1:** Proposed minimal set of biomarkers to monitor age-related changes in chronic inflammation and responsiveness to bacterial and viral challenges.

Endogenous	*E*. *coli* Stimulation	HSV-1 Stimulation
↓ G-CSF (EDTA)	↓ G-CSF (EDTA)	↓ G-CSF (EDTA)
↑ PGE_2_ (EDTA)	↓PGE_2_ (heparin)	↑PGE_2_ (heparin)
↑ CRP (heparin)	IL-6 (heparin)	↓ IFNα (heparin)
IL-6 (heparin)		IL-6 (heparin)

The anti-coagulant recommended for each marker is shown in brackets. The difference in G-CSF with age correlates with MIP1α and IL-17 so it is not necessary to test for these latter 2 markers. Although IL-6 (heparin) does not appear to vary with age in response to *E*. *coli* or HSV-1 in our healthy volunteers it is still informative because of its large person-to-person variation. Since this variation correlates relatively well with that seen with IL-1β, TNFα, MIP1α and IL-8 (see **S2 Table**), IL-6 is sufficient for these cytokines and chemokines. Arrows indicate if levels increase or decrease with age.

## Supporting information

S1 Fig **EDTA-collected blood samples from 120 volunteers were stimulated with *E*. *coli* (panel A) or HSV-1 (panels B & C) and analyzed for G-CSF levels (panels A & B) or IFNα levels (panel C)**. ● = 20–34 years old, ■ = 35–49 years old, ▲ = 50–77 years old. * indicates a statistically significant difference (P <0.05) between cohorts.
(TIF)

S2 Fig Control, *E*. *coli* and HSV-1 stimulated cytokine and chemokine levels from 41 healthy volunteers, each with a unique identifying symbol. (TIF)

S3 Fig Control, *E*. *coli* and HSV-1 stimulated PGE_2_ levels as well as anti-CD3 + anti-CD28 stimulated PBMC levels of proliferation and IFNγ production from 41 healthy volunteers. (TIF)

S1 Table Pearson correlation matrix between age, BMI and zero time cytokine/chemokine levels of 41 people. (TIF)

S2 Table Pearson correlation matrix between age, BMI and *E*. *coli*-stimulated cytokine/chemokine levels of 41 people. (TIF)

S3 Table Pearson correlation matrix between age, BMI and HSV-1-stimulated cytokine/chemokine levels of 41 people. (TIF)
