# Supplementary figures and images for: Effect of age on chronic inflammation and responsiveness to bacterial and viral challenges
Source: PLoS One. 2017 Nov 29;12(11):e0188881. doi: 10.1371/journal.pone.0188881 (PMC5706672; doi:10.1371/journal.pone.0188881)

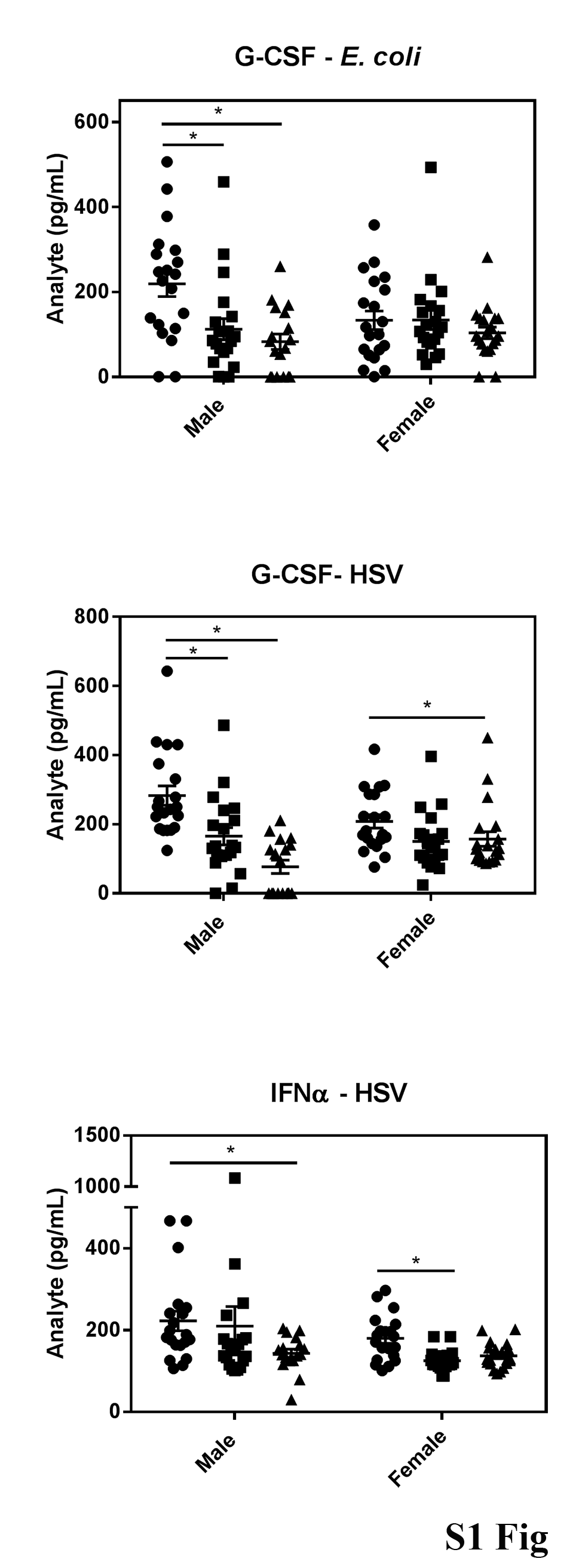

Supplement: S1 Fig — EDTA-collected blood samples from 120 volunteers were stimulated with E. coli (panel A) or HSV-1 (panels B & C) and analyzed for G-CSF levels (panels A & B) or IFNα levels (panel C). ● = 20–34 years old, ■ = 35–49 years old, ▲ = 50–77 years old. * indicates a statistically significant difference (P <0.05) between cohorts. (TIF) [file pone.0188881.s001.tif]

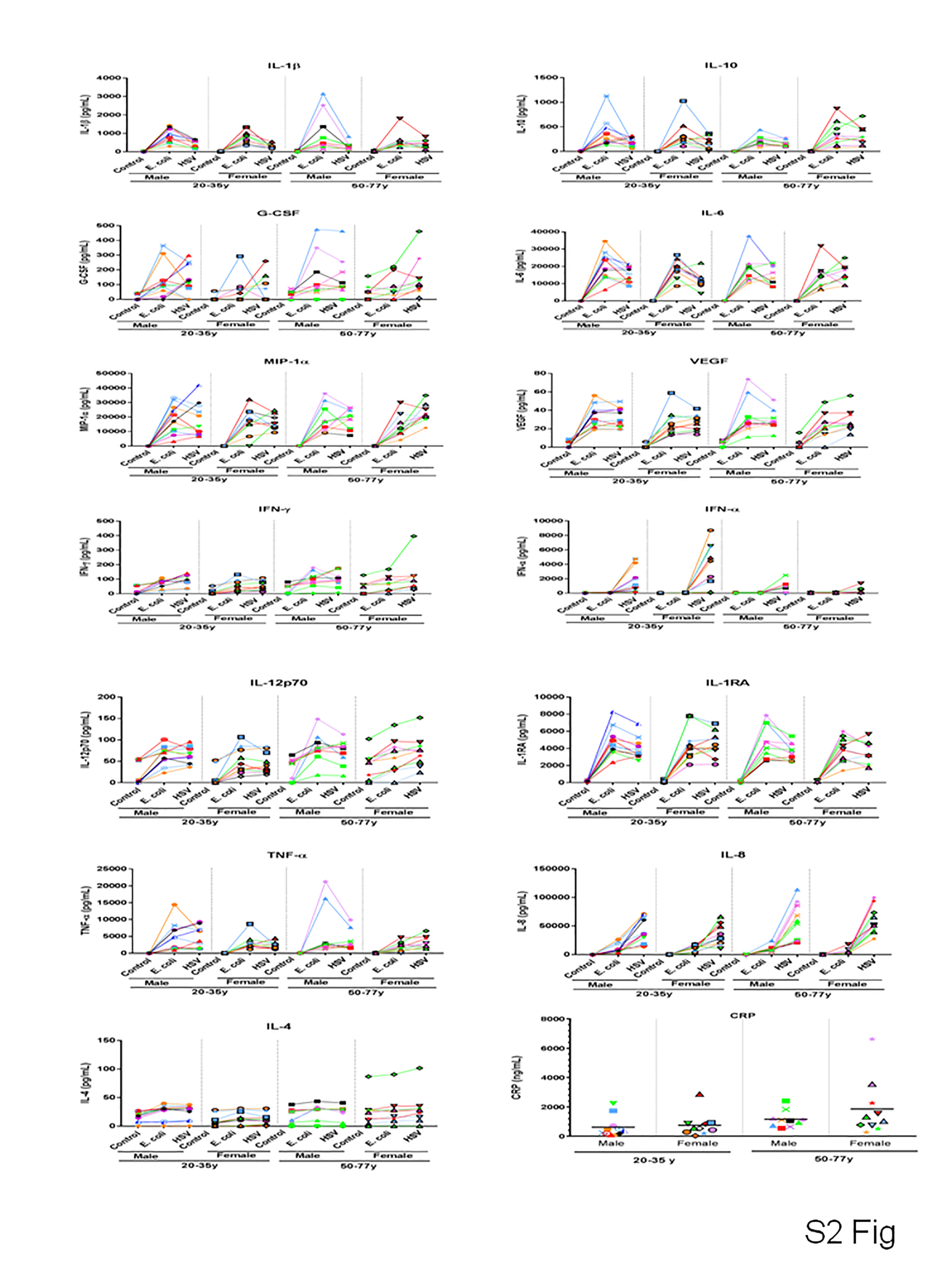

Supplement: S2 Fig — (TIF) [file pone.0188881.s002.tif]

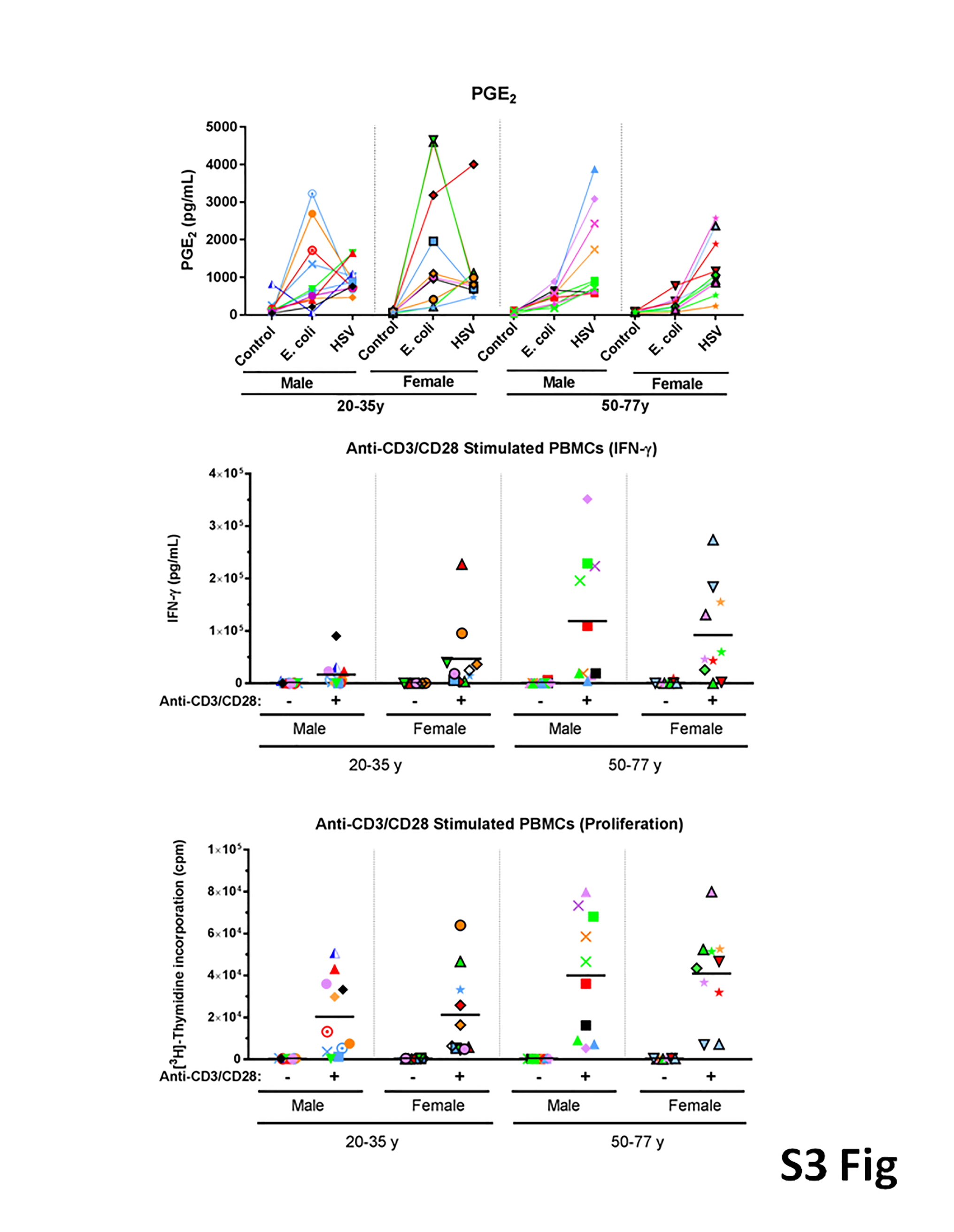

Supplement: S3 Fig — (TIF) [file pone.0188881.s003.tif]

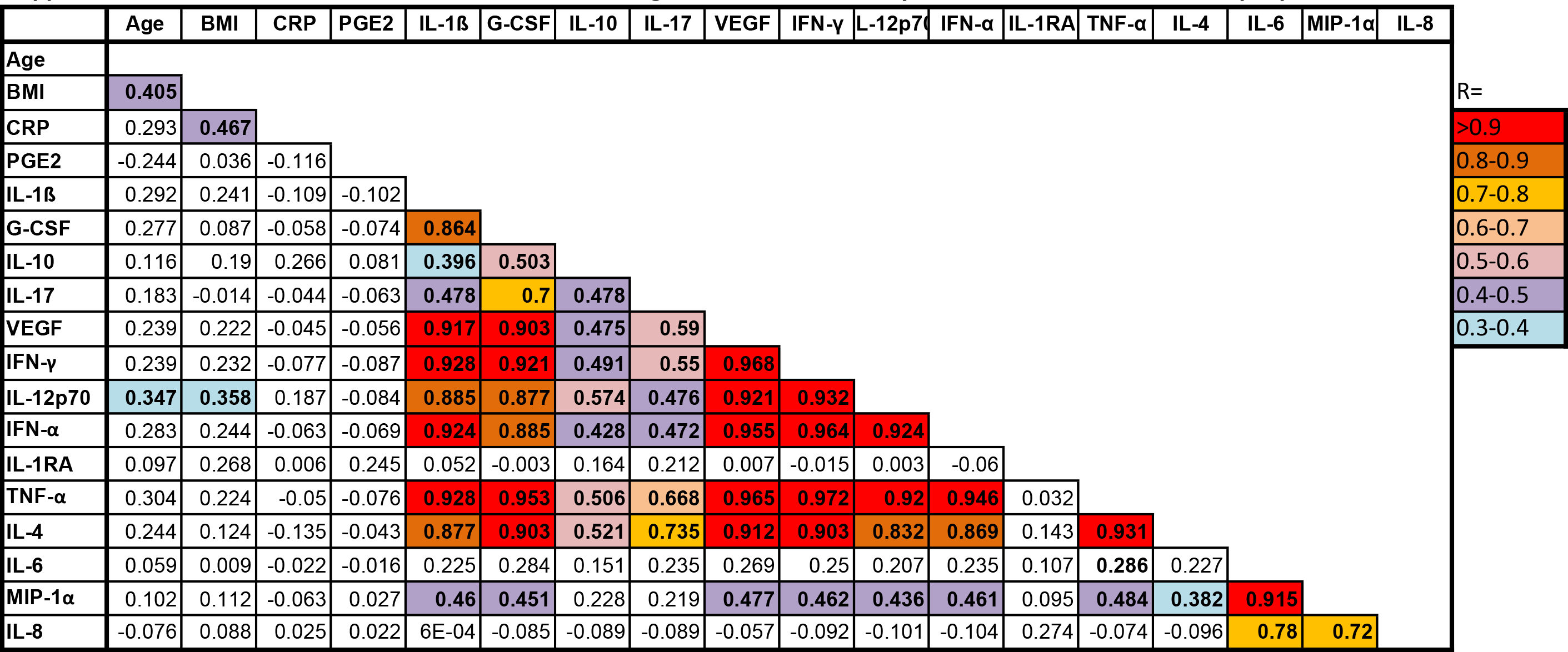

Supplement: S1 Table — (TIF) [file pone.0188881.s004.tif]

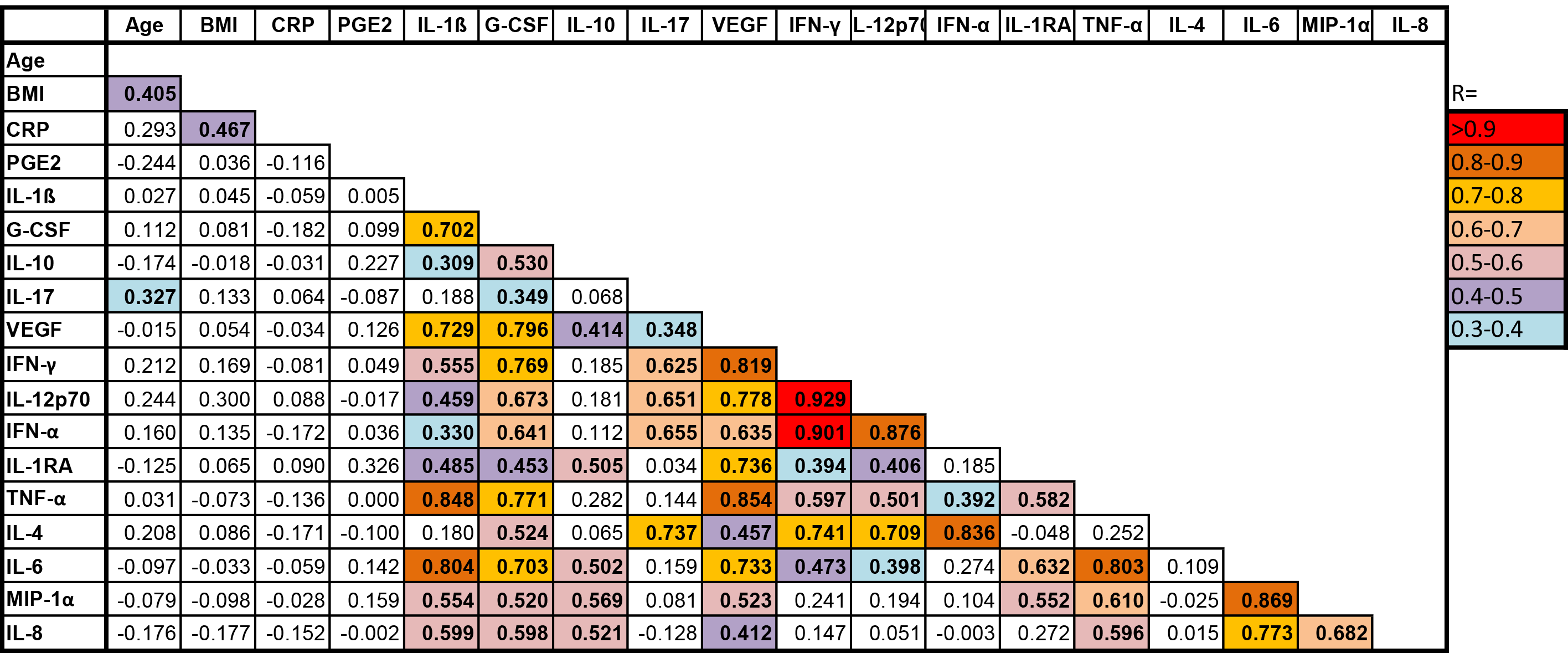

Supplement: S2 Table — (TIF) [file pone.0188881.s005.tif]

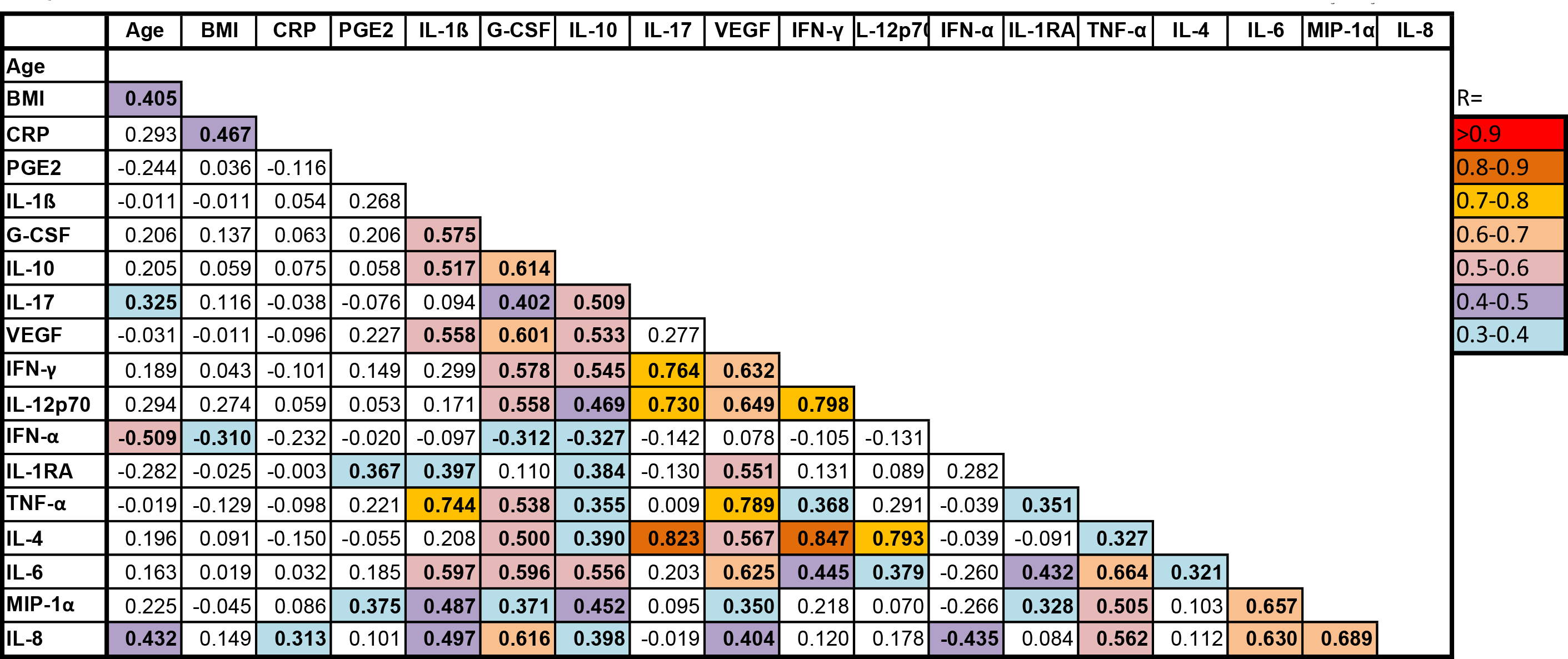

Supplement: S3 Table — (TIF) [file pone.0188881.s006.tif]
